# Harnessing Poly(9,9-dialkylfluorene-alt-benzothiadiazole) for Circularly Polarized Electroluminescence: Advances and Perspectives

**DOI:** 10.3390/ma19061224

**Published:** 2026-03-20

**Authors:** Mariacecilia Pasini, Umberto Giovanella

**Affiliations:** Istituto di Scienze e Tecnologie Chimiche “Giulio Natta” (SCITEC), Consiglio Nazionale Delle Ricerche (CNR), Via A. Corti 12, 20133 Milano, Italy; mariacecilia.pasini@cnr.it

**Keywords:** poly(9,9-dialkylfluorene-alt-benzothiadiazole), F8BT, PFBT, chirality, CP-OLEDs, chiral inducers

## Abstract

Circularly polarized (CP) organic light-emitting diodes (CP-OLEDs) have attracted considerable attention due to their promising applications in next-generation display systems, optical data transmission, and quantum computing, and their potential roles in medical devices. Achieving efficient and tunable CP emission remains a significant challenge, prompting the development of various strategies that leverage organic semiconductors. Notably, certain classes of materials now consistently deliver CP polarization at levels suitable for technological applications. Among these, conjugated polymers, particularly the copolymer poly(9,9-dialkylfluorene-alt-benzothiadiazole) (PFBT), stand out for their exceptional optoelectronic properties, ease of processing, and adaptability to produce CP emission. PFBT has played diverse roles within CP-OLED devices, enabling innovative architectural solutions. This review explores principal strategies for integrating PFBT into CP-OLED architectures, drawing upon findings from the recent scientific literature. By consolidating current knowledge and identifying unresolved issues, this work aims to inspire further research into the development of solution-processable, high-performance and tunable CP-OLEDs based on PFBT and conjugated polymers in general.

## 1. Introduction

Organic light-emitting diodes (OLEDs) exhibiting circularly polarized (CP) luminescence (CPL) have become a prominent research focus across multiple disciplines owing to their considerable potential in applications such as three-dimensional displays, spintronics, optical data storage, quantum computing, and medical devices [1,2,3,4]. Since the initial report of CP OLEDs (CP-OLEDs) by Meijer et al. in 1997 [5], there has been growing interest in this field, leading to the development of various CP electroluminescence (EL) emitters [6]. These emitters include chiral conjugated polymers [7,8], chiral organometallic complexes [9,10,11,12], and chiral organic small molecules [13,14,15,16], as well as chiral TADF-active materials [17,18], together with inorganic [19] and hybrid/inorganic compounds such as perovskites [20,21].

**Figure 1:**
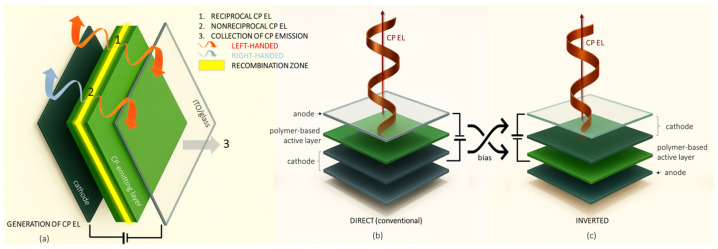
Typical CP-OLED architecture with recombination zone (RZ) for radiative excitons and reciprocal and nonreciprocal CP EL (**a**). Conventional (**b**) and inverted (**c**) device architecture.

CP emission is generally associated with chiroptical activity, which is quantitatively described by the dissymmetry factor, or g-factor, as g = 2(I_L_ − I_R_)/(I_L_ + I_R_), where I_L_ and I_R_ represent the intensities of left-handed and right-handed intensity of CP emission, respectively. The g-factor serves as a measure of the chiroptical response for various optical phenomena, including absorption (g_abs_) and photoluminescence (g_PL_), with possible values ranging from +2 (fully left-handed) to −2 (fully right-handed) CPL. When these chiral optoelectronic materials are integrated into OLEDs, they can directly generate CP EL, which is characterized by the g_EL_ parameter [5]. The most common type of CP EL emission is reciprocal, occurring when a chiral chromophore emits CPL with the same sign in both directions (forwards and backwards) (Figure 1a). However, in chiral assemblies, nonreciprocal CP EL can also be observed (Figure 1a) with oppositely handed luminescence from the front and back face of the sample. For a more in-depth description of circular polarization of PL emission in chiral systems and how CP EL (either reciprocal or nonreciprocal) originates in OLEDs, we refer the reader to more specialized reviews [22,23,24,25].

To more effectively assess the performance of CP-OLEDs, a practical metric known as the Q-factor [26], defined as EQE × |g_EL_|, has been recently introduced, where EQE denotes the external quantum efficiency of the OLED. This metric is analogous to the commonly used CPL brightness parameter, B_CPL_ = ɛ × Φ_f_ × |g_PL_|/2 [27], where ɛ is extinction coefficient and Φ_f_ is PL quantum yield of the CP emitter.

Achieving both strong polarization and high EQE is challenging, as the selection of emissive species and their respective emission mechanisms that enhance one property often compromise the other. To address this intrinsic trade-off, researchers have explored a variety of strategies [28], including the molecular engineering of emissive materials to enhance chiral organization, modifications to device architecture to optimize CP emission out-coupling, and the use of advanced host–guest systems that promote both efficient charge transport and strong chiral interactions. Through these approaches, the goal is to design CP-OLEDs that simultaneously deliver robust circular polarization and maintain high EQE, hence high Q-factors.

Conjugated polymers are particularly attractive for OLEDs in general, and specifically for CP-OLEDs [22]. Two principal approaches have been developed to introduce chirality into electroluminescent polymer-based devices: (A) blending achiral conjugated polymers with chiral small-molecule additives with high helical twisting power (HTP) (hereafter A-strategy), and (B) directly functionalizing conjugated polymer backbones with chiral sidechains (B-strategy).

**Figure 2:**
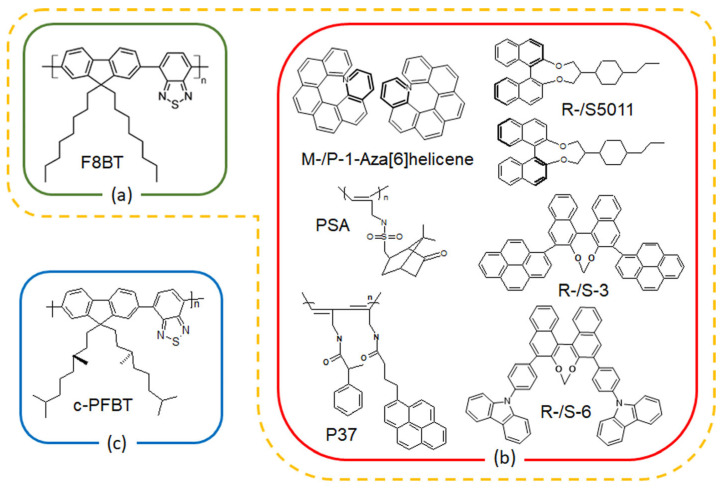
(**a**) Chemical structure of achiral F8BT and (**b**) small molecules and polymers used as chiral inducers in the blends, and (**c**) F8BT with chiral sidechains (c-PFBT).

From a material design standpoint, conjugated polyfluorene derivatives, particularly poly(9,9-alkylfluorene-alt-benzothiadiazole (PFBT), demonstrate a very high degree of g_EL_ [29,30] when blended with chiral molecules. The PFBT, also referred to as F8BT (Figure 2a) in this review when bearing octyl side chains on the fluorene moiety, was a groundbreaking conjugated polymer employed in the early development of polymer OLEDs for efficient green light emission, enabling solution processing and high performance, even in flexible or inverted device architectures. This polymer has been extensively used both as a neat emitter [31,32] and as a host matrix for guest chromophores, leveraging Förster Resonance Energy Transfer (FRET) mechanisms for color tuning, and even serving as a charge regulating layer [33]. F8BT-based systems have enabled diverse fluorescent CP-OLED device architectures, influencing not only the degree of circular polarization but also charge balance, emission efficiency, and operational stability.

Despite the rapidly expanding literature [22,23,24,25,34,35,36], a comprehensive and systematic review that simultaneously addresses both the material-level design of PFBT-based chiral systems and their incorporation into device architectures and performance metrics is still lacking.

Research into polyfluorene-based CP-OLEDs demonstrates that F8BT serves effectively as a host for the dispersion of a wide range of guest dyes. Helical aromatic compounds, such as helicenes [37,38,39], axially chiral binaphthyl derivatives [29,40], and chiral cyclophanes [41], have been identified as efficient HTP additives (Figure 2b) for the realization of CP-OLEDs (A-strategy) [42,43]. In these co-assemblies, the chiral molecule induces supramolecular arrangements in the host polymer [44], resulting in CP emission, with the polymer itself being the source of CP emission. There are also examples where the chiral molecule acts as the CP-emitting species; however, this approach is currently limited to the incorporation of chiral emitters, typically metalorganic or lanthanide complexes, into polymer hosts such as PVK [9] and is therefore not discussed further in this review. An alternative strategy involves the direct attachment of enantiopure sidechains to the fluorene units in fluorene–benzothiadiazole copolymers (c-PFBT, Figure 2c), resulting in strong chiroptical effects (B-strategy) [30,45].

Generally, in both strategies, the characteristics of conjugated polymers (i.e., molecular weight) and thin-film processing conditions, including solvent selection, temperature and annealing time, play critical roles in inducing and tuning the chiroptical properties of these materials, in both film and device applications. These factors directly affect the magnitude, sign, and reproducibility of the chiroptical response, making their careful consideration essential for the rational design of CP-OLEDs. For instance, F8BT undergoes notable structural changes upon annealing, which in turn influence its optoelectronic properties. Previous research has shown that thermal annealing affects the packing arrangement, optoelectronic behavior, and charge transport in F8BT films, with these effects being closely linked to the polymer’s molecular weight (Mn, Mw) [46,47]. Most studies employ Mw values between approximately 9 and 255 kDa. However, very high Mw can hinder liquid crystalline ordering, and a Mw of about 35 kDa is most frequently selected for CP-OLED production [48].

Notably, the influence of processing parameters extends beyond polarization intensity and for F8BT, not only does the degree of circular polarization depend on the film’s thickness, but an unusual dependence of the polarization’s sign on the thickness of the F8BT film has also been observed [49].

In view of these observations, this review will place particular emphasis on identifying and discussing processing- and morphology-related parameters wherever reported, with the goal of deepening the understanding of their role in determining chiroptical properties and device performance. The current lack of an integrated perspective that connects chiral material design with processing-dependent film structure and device performance in PFBT-based CP-OLEDs is addressed. To this end, we critically examine the two main approaches for introducing chirality, small-molecule additive induction (Strategy A) and chiral side-chain engineering (Strategy B), and extend the analysis beyond molecular design by evaluating how thin-film preparation parameters, such as solvent conditions, thermal annealing, and film thickness, directly influence chiroptical behavior and OLED characteristics. By establishing these links, we provide a uniquely comprehensive overview of the development of F8BT-based CP-OLEDs.

Importantly, this review focuses exclusively on studies in which PFBT (F8BT) has been integrated into OLED configuration leading to CP EL. Although numerous reports discuss the chiral organization and CPL of F8BT-based thin films, the translation of these material-level advances into fully operational CP-OLEDs presents a distinct and non-trivial step, which constitutes the primary scope of the present work. The two strategies (A and B) are discussed in detail in the following sections, and we intend to conclude by outlining future perspectives for the advancement of this material class in light of the most critical parameters identified across the literature.

The OLED architectures encountered along this review differ in electrode placement: direct (conventional) devices have a transparent anode and a reflective cathode, while inverted one uses transparent cathode and reflective anode (Figure 1b,c).

## 2. Overview of Recent Advances

### 2.1. F8BT Achiral Emitter Doped with a Chiral Inducer (Strategy A)

The first approach is the most commonly used and has been implemented by numerous research teams. It focuses on introducing, i.e., blending, a chiral inductor, such as a chiral small molecule or polymer, into the achiral F8BT emitter. In this strategy, the chiral inductor itself does not generally contribute to light emission but instead generates a chiral environment within the F8BT matrix, thereby enabling CP emission. This technique is considered highly practical for transforming an achiral conjugated polymer into a CP-emitting material. Recent studies highlight the importance of forming chiral supramolecular structures through intermolecular interactions for efficient chirality transfer. Notably, employing chiral helicene or binaphthyl derivative inducers has led to substantial improvements in CP EL while preserving the inherent emission characteristics of F8BT. The effectiveness of this method is largely determined by the miscibility and compatibility between F8BT and the chiral inductor, as well as the thermal stability of the resulting blend. The explanation for the often not obvious behavior observed in related CP devices remains to be clarified, and to this aim, current research efforts continue to integrate morphological, spectroscopic, and electronic analyses of thin films and devices with theoretical investigations.

**Figure 3:**
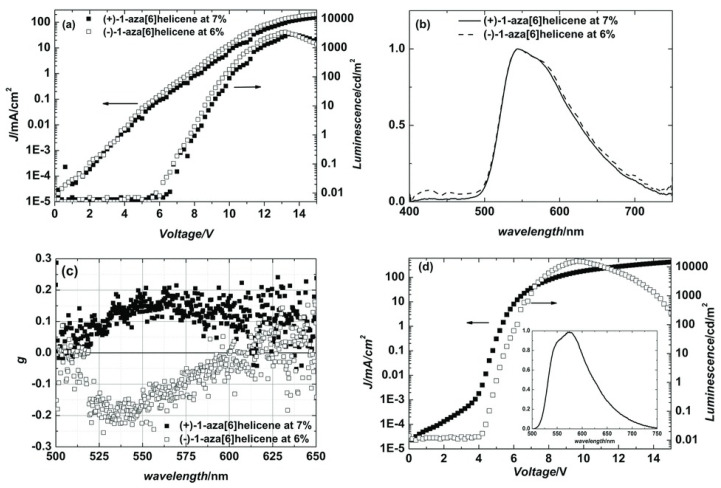
(**a**) Current density (circles) and luminance (squares) with applied voltage (J-L-V curves). (**b**) EL spectra and (**c**) g_EL_ of the CP-OLED device containing F8BT doped with 7% (by weight) of (+)-1-aza[6]helicene (solid symbols) and 6% (by weight) (−)-1-aza[6]helicene (open symbols). (**d**) J-V (black square symbols) and L-V curves (empty square symbols) of an undoped (reference) F8BT OLED with EL spectrum as the inset. Reproduced with permission from Ref. [37]. Copyright 2013 Wiley-VCH GmbH.

Fuchter and co-workers [37] proposed the direct generation of pronounced CP EL by doping the achiral F8BT with 7 wt% of the chiral aromatic molecule aza[6]helicene (or aza[6]H, Figure 2b) in toluene; however, no information regarding the Mw of the polymer was reported. The aza[6]H is an intrinsically helical, conjugated molecule composed of spirally fused carbocyclic or heterocyclic rings, which impart inherent chirality [50,51,52]. These HTP molecules can be resolved into their right-handed and left-handed enantiomers, each exhibiting pronounced chiroptical characteristics, including substantial optical rotatory power and strong circular dichroism (CD) [53,54]. Notably, no thermal treatment is needed for the F8BT:aza[6]H film. This pioneering study demonstrated a proof-of-concept, single-layer, un-optimized CP-OLED, featuring a g_EL_ factor as high as 0.2 alongside a bright emission of 3000 cd/m^2^ (Figure 3a,c) from devices incorporating each of the two enantiomers, with an efficiency of 1.1 lm/W.

This approach has emerged as one of the most effective methods for achieving CP EL with large g_EL_ values and has catalyzed further research, including the exploration of various helicene derivatives and other chiral inducers for enhancing CP emission.

**Figure 4:**
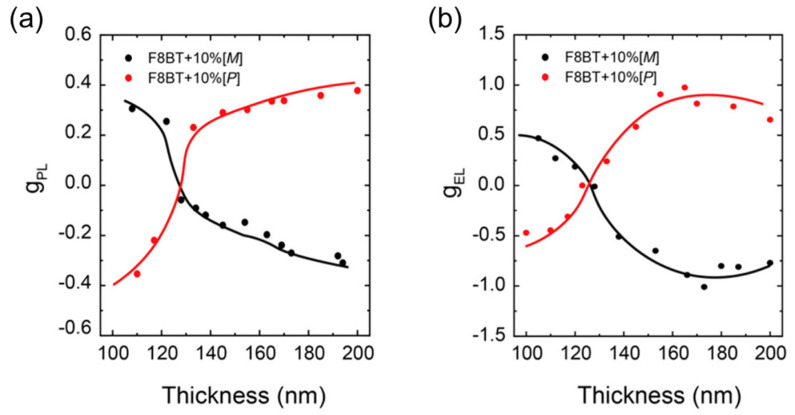
(**a**) *g*_PL_ as a function of film thickness, extracted from the emission band maximum (λ = 546 nm). (**b**) *g*_EL_ as a function of active layer thickness, extracted from the emission band maximum for device structure: ITO/PEDOT:PSS/TFB/F8BT + 10% [*M*]/[*P*]-aza[6]H/Ca/Al. Adapted with permission from Ref. [49]. Copyright 2019 American Chemical Society.

Wan et al. [49] revealed a noteworthy chiroptical phenomenon: the apparent dissymmetry in CP EL and CPL of F8BT:aza[6]H film can be modulated solely by adjusting the thickness of the active layer (Figure 4). Remarkably high g-factors were observed for both left- and right-handed CP light when using a single-handed (enantiopure) aza[6]H additive in F8BT, without requiring an alignment layer. The optimal performance was achieved at a helicene doping of 10 wt%, using F8BT with a Mw of approximately 31 kDa, and films prepared from toluene solutions. The dissymmetry for both polarization states could be tuned by varying the film thickness: thin films (110 nm) yielded g_EL_ = 0.51, while thick films (160 nm) produced g_EL_ = −1.05. Additionally, device performance significantly surpassed previous reports, achieving simultaneous improvements in both efficiency (4.0 cd/A) and luminance (8000 cd/m^2^).

The thickness dependence was attributed to the interplay between localized CP emissions arising from molecular chirality and the amplification or inversion of CP emissions [55] through the chiral medium. By manipulating the active layer thickness and device architecture, the study provides valuable insights into the mechanisms driving CP luminescence and high performance in CP-OLEDs, while highlighting new opportunities for the design of CP photonic devices.

Introducing hole transport (HT)/hole injection (HI) materials and electron transport (ET)/electron injection (EI) materials (blended into the active layer or as interlayers or charge regulating layers at the electrode interfaces) [56] in a device structure is a highly fascinating approach to increase and balance the carrier injection or transport ability and optimize the performance of CP-OLEDs. In fact, so far, there has been no report about CP-OLEDs from the blends of achiral fluorescent polymers and chiral dopants with excellent or well-balanced carrier mobility. In this view, Yan et al. [57] proposed a method to enhance charge injection and achieve charge balancing in CP optoelectronic devices by introducing a small quantity (about 1.5 wt%) of a chemically modified electrochemical doping agent, 1-dodecly-3-imidazolium hexafluoro-phosphate (EDA). The EDA, featuring a long cationic alkyl chain, imparts pronounced hydrophobicity and facilitates stable electrochemical doping in achiral F8BT within F8BT:aza[6]H blends (10%) using a low Mw F8BT (9 kDa) processed from toluene solutions and annealed at 140 °C for 10 min. This modification promotes efficient charge injection and improves charge balance, which collectively enhance device performance. Notably, the devices maintain a high degree of polarization, with g_EL_ values of 0.50 at 580 nm and −0.65 at 525 nm within the ITO/PEDOT:PSS/TFB/EML/Ca/Al architecture (where TFB is Poly(9,9-dioctylfluorene-alt-N-(4-sec-butylphenyl)-diphenylamine) and acts as an HT layer). These results suggest that EDAs offer a straightforward method for boosting the performance of CP optoelectronic devices.

In contrast to previous studies utilizing helicenes, and to enhance carrier mobility at the molecular design level, in 2020, Zhang et al. [58] synthesized a pair of 1,1′-binaphthalenyl-based enantiomers (R-/S-3) by integrating pyrene groups (Figure 2b). To examine the chiral induction capabilities of R-/S-3 dopants on F8BT (molecular weight Mw = 10–100 kDa from Macklin Inc., Shanghai, China), CP-OLEDs were fabricated using various mixing ratios of R-/S-3 (5–25 wt%) with F8BT in chlorobenzene (total blend concentration: 16 mg mL^−1^). The thermal annealing temperature of emitting layer blends was 140 °C for 10 min with the device structure ITO/PEDOT:PSS/F8BT:R-/S-3/TPBI/Ca/Ag (where TPBI is 2,2′,2″-(1,3,5-benzinetriyl)tris(1-phenyl-1-H-benzimidazole) and acts as EI layer). Devices with relatively thin emissive layers (~35 nm) exhibited good EQE up to 1.4%, high luminance exceeding 20,000 cd/m^2^, low turn-on voltage (V_ON_, 4.1 V), and above all a g_EL_ approaching 10^−2^. The best performance was obtained in F8BT + 10wt%R-/S-3-based devices and was attributed to the well-balanced charge carrier transport ability and homogeneous film formation. The following year, Zhang et al. [40] synthesized chiral binaphthyl derivatives (Figure 2c) as CP EL inductors by modifying 1,1′-binaphthol (BINOL) with various alkyl chains or functional groups. These changes allowed the fine-tuning of their chiral induction on achiral F8BT in doped films. Notably, R/S-6, with its planar rigid structure, produced strong CPL signals and, thanks to its excellent carrier mobility, was selected for constructing CP-OLEDs (F8BT + 5–25 wt% R/S-6). Devices using these blends exhibited low V_ON_ < 4.5 V, high luminance (>10,000 cd/m^2^), and a maximum |*g*_EL_| of 1.86 × 10^−2^, with a Q-factor of ~10^−5^. No emission from R/S-6 was seen in the EL spectra, indicating it serves as an effective carrier transport material without altering F8BT’s emission profile.

**Figure 5:**
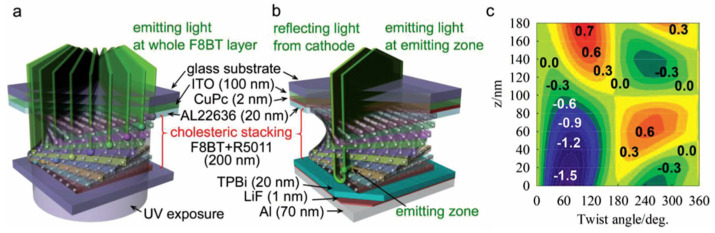
Schematic diagrams of the twisted stacking of rigid rods describing F8BT molecules in a sublayer for the (**a**) CP PL and (**b**) CP EL calculations. (**c**) Contour map of the calculated |g_EL_| as a function of the twist angle and the location of the RZ (z). The numbers are values of the |g_EL_| factor, with colors ranging from blue to green, yellow and red to indicate increasing magnitude. Adapted with permission from Ref. [29]. Copyright 2018 Wiley-VCH GmbH.

Lee et al. [29] employed R5011 (Figure 2b) as the chiral dopant. CP-OLEDs were fabricated by blending F8BT (Mw = 70 kDa, from Lumtec), having a nematic liquid crystalline phase over T = 125 °C, with varying ratios of R5011 (up to 50%) in toluene as the emitting layer in the device configuration: ITO/CuPC/PI/F8BT:R5011/TPBI/LiF/Al. In this architecture, copper phthalocyanine (CuPC) and PBI function as the HI layer and HB layer, respectively, while a polyimide PI serves as a rubbed alignment layer for the emitting layer (Figure 5).

No CP emission was observed unless the system underwent thermal treatment above the glass transition temperature (T_g_) of F8BT. Despite thermal treatment, uniform twisted alignment was not achieved, probably because R5011 did not induce a helical molecular conformation in the host material as helicenes have done in previous studies [37]. Instead, R5011 promoted a twisted (cholesteric) stacking of the host material at the macroscopic scale, attributed to its high HTP. This finding suggests that twisted stacking may play a more significant role than helical molecular conformation in generating CP light.

The g_PL_ and g_EL_ values were markedly enhanced, reaching values of −0.72 and −1.13 at λ = 546 nm, attributed to linearly polarized (LP) light generated by the F8BT layer (aligned by PI film) being converted into CP emission as it traverses the twisted stacking of the birefringent F8BT. As a result, by employing a theoretical framework based on Mueller matrix analysis and Stokes parameters, the position and extent of the recombination (emission) zone (RZ), the film’s birefringence, and the degree of linear polarization emerge as critical factors for CP EL generation. When RZ is situated near the interface with the TPBI, CP EL is considerably amplified.

The handedness of the CP emission reverses depending on the chirality of the dopant, and the formation of a perfect monodomain alignment is not required. Bright CP EL emission, with luminance up to 4000 cd/m^2^ and an efficiency of 4.46 cd/A, was recorded for CP-OLEDs featuring an active layer thickness of 200 nm.

The use of interlayers and an inverted device architecture was explored by Wan et al. [59] with an active layer of F8BT (Mw = 31 kDa, Cambridge Display Technology Ltd., Godmanchester, UK) with 10 wt% [M]-aza[6]H in toluene. To induce chirality within the active layer, the devices were annealed at 140 °C for 10 min in a nitrogen-filled glovebox. At this temperature, racemization of aza[6]H is negligible, but it is shown to aggregate at the HT layer/active layer interface, compromising device performance in CP-OLEDs. The inverted device geometry (Figure 1c) circumvents this problem, enabling the concurrent achievement of high efficiency and pronounced dissymmetry, two key performance metrics that have previously been challenging to realize together. Their devices exhibit a current efficiency of 16.4 cd/A, a power efficiency of 16.6 lm/W, a peak luminance exceeding 28,500 cd/m^2^, and a notable g_EL_ of 0.57. The inverted device geometry and incorporation of a tris(4-carbazoyl-9-ylphenyl)amine (TCTA) HT/HI layer led to balanced charge carrier injection and diminished charge carrier trapping, which in turn optimized the location of the RZ within the active layer to minimize unfavored nonradiative decay pathways. Furthermore, the study reveals that the handedness of the emitted light is sensitive to the device architecture. Specifically, conventional and inverted CP-OLEDs fabricated with the same enantiomer of an emissive chiral F8BT:[M]-aza[6]H-blend material produce CP EL of opposite handedness. This phenomenon, termed nonreciprocal or anomalous CP EL, is further demonstrated by observing that CP EL emitted from the front and back of a semitransparent device exhibits opposite handedness. The direction of the charge current influences the directionality of the chiroptical response due to the polarizing effect that the chiral medium has on the charge carriers, outweighing the intrinsic chiroptical behavior of the chromophore. The study is currently limited to the F8BT:[M]-aza[6]H-blend active layer, and the practical exploitation of nonreciprocal CP EL effects in the development of high-performance CP-OLEDs remains quite unresolved [60]. However, anomalous CP EL may permit substantially greater g_EL_ values than those attainable through g_PL_.

There are some examples where the supramolecular organization induced in F8BT by a chiral molecule can, via an energy transfer mechanism, enable polarized emission from an achiral chromophore further dispersed within the matrix, so that it is the achiral chromophore which emits CP EL.

**Figure 6:**
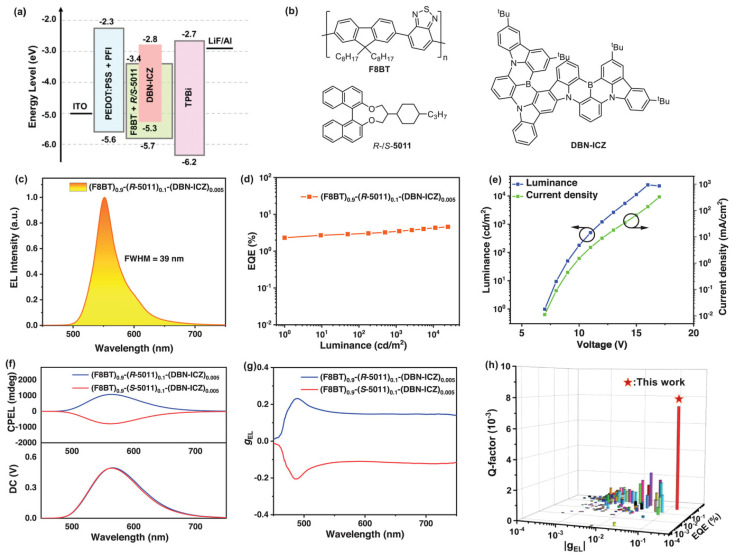
(**a**) Energy diagram and device structure of CP-OLEDs based on co-assembled films F8BT:R-5011:DBN-ICZ. (**b**) Molecular structures of emitting-layers. (**c**) EL spectra. (**d**) EQE-luminance characteristics. (**e**) J-V (green square symbols) and L-V curves (blue square symbols). (**f**) CP EL spectra. (**g**) g_EL_ values as a function of emission wavelength. (**h**) The Q-factor values of reported CP-OLEDs (with red star to indicate the record value achieved for F8BT(0.9):R/S-5011(0.1):DBN-ICZ(0.005) device). Reproduced with permission from Ref. [26]. Copyright 2024 Wiley-VCH GmbH.

Recently, Guo et al. [26] reported the development of ternary chiral co-assemblies combining F8BT (Mn = 22.4 kDa, Mw/Mn = 2.70 from Xi’an Yuri Solar, Xi’an, China) with R/S-5011 to construct chiral assembled host structures featuring high g_PL_ values upon thermal annealing. For efficient FRET, the achiral TADF molecule, DBN-ICZ (Figure 6b), was selected as the energy acceptor due to its absorption spectrum matching the emission of the chiral co-assemblies F8BT:R/S-5011. The pronounced g_PL_ values observed in these chiral co-assemblies are primarily attributed to the long-range ordered stacking of chromophores, which facilitates chiral exciton coupling. Importantly, these chiral co-assemblies F8BT:R/S-5011:DBN-ICZ were utilized as emitting layers in CP-OLEDs. The chiral co-assemblies films prepared in toluene and containing different DBN-ICZ doping concentrations displayed CD silence before thermal annealing. After thermal annealing at 140 °C for 10 min, the co-doped films F8BT(0.9):R/S-5011(0.1) exhibited intense CD signals. The annealing temperature was optimized according to the T_g_ of F8BT and the g_PL_ values of the co-assembled films. The T_g_ of F8BT was determined to be 130 °C, and since the annealing temperature must generally exceed T_g_ to activate the assembly process, analysis of g_PL_ values at various annealing temperatures indicated that the optimal annealing temperature was 140 °C. The optimized devices after thermal treatment, with the ternary chiral co-assemblies F8BT(0.9):R/S-5011(0.1):DBN-ICZ(0.005), exhibited yellow EL, peaking at 552 nm (Figure 6c), with a maximum EQE of 4.6% (Figure 6d) and strong CP EL signals (Figure 6f), reaching a g_EL_ value of up to 0.16 (Figure 6g). Notably, the Q-factor for the F8BT:R/S-5011:DBN-ICZ-based devices reached ~7 × 10^−3^, representing the highest value reported for CP-OLEDs to date (Figure 6h). This ternary co-assembly strategy offers a promising avenue for fabricating CP-OLEDs that balance high EQE and large g_EL_ with high brightness.

**Figure 7:**
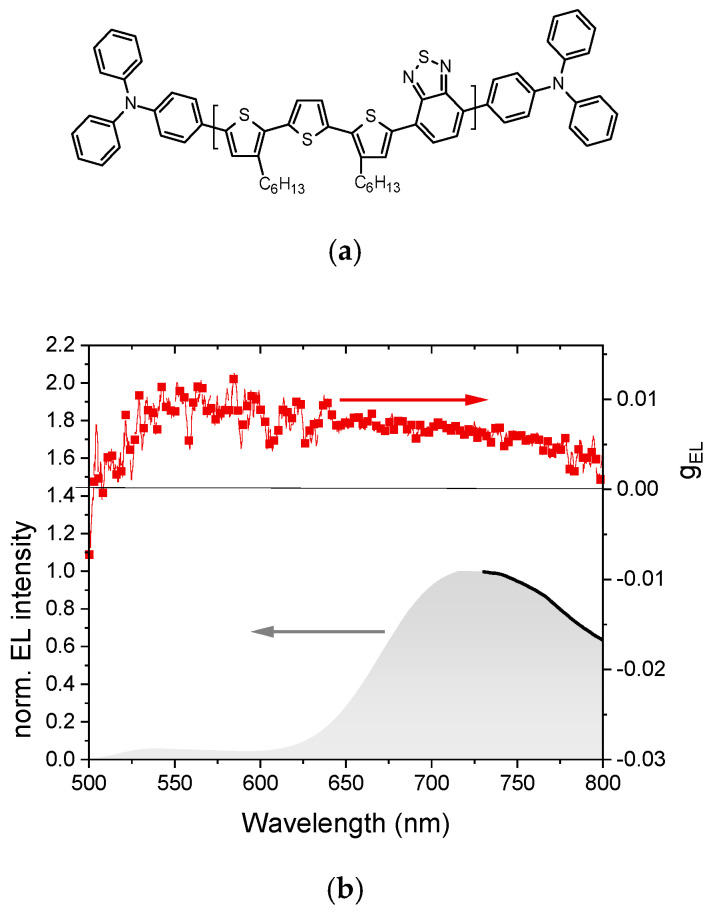
(**a**) Chemical structures of 3TBT-TPA; (**b**) EL (gray filled area) and g_EL_ (red square symbols) of ITO/PEDOT:PSS/F8BT:3TBT-TPA:R5011/LiF/Al device. Adapted with permission from Ref. [48]. Copyright 2025 Wiley-VCH GmbH.

Squeo et al. [48] utilized the same F8BT:R5011 blend as a chiral host matrix for an achiral conjugated polymer guest, thereby enabling the CP emission in the near-infrared (NIR) region. The conjugated copolymer, 3TBT-TPA, consists of a terthiophene (3T) and benzothiadiazole (BT) backbone, end-capped with triphenylamine (TPA) units (Figure 7a). By employing a co-assembly approach, chirality was induced in the F8BT:3TBT-TPA blend through the incorporation of the chiral inducer R5011. A F8BT:3TBT-TPA:R5011 ternary blend film, prepared in toluene with a 10:1:2 mass content ratio, was thermally treated at 140 °C for 30 min to promote chiral self-organization. This strategy resulted in CP EL within the 500–800 nm spectral range, with dissymmetry factors of approximately 5 × 10^−3^ for the NIR emission from 3TBT-TPA (Figure 7b). Notably, this work represents the first reported CP-NIR-OLEDs that also feature direct CP EL from a conjugated polymer. Furthermore, the observed inversion in the sign of CP EL compared to CP PL underscores the intricate interplay of factors affecting device performance, such as circular self-extinction in the emissive layer, the spatial location of the radiative exciton RZ, and the overall device architecture.

**Figure 8:**
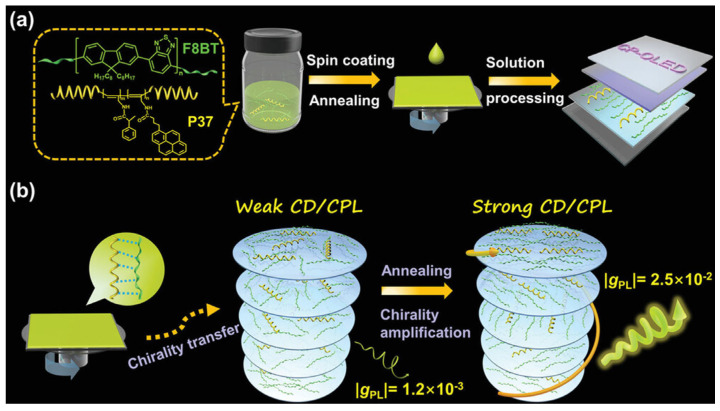
(**a**) Preparation of the chiral helical polymer-induced CP-OLEDs. (**b**) The proposed mechanism for chirality transfer and chirality amplification within chiral emitting layer. Reproduced with permission from Ref. [61]. Copyright 2023 Wiley-VCH GmbH.

Current research in chirality induction-type CP-OLEDs has predominantly focused on systems based on small molecules as chiral inductors. Wang et al. [61] proposed chiral helical polymers for F8BT-based CP-OLED construction. In contrast to chiral small molecules, chiral helical polymers possess higher-order chirality and exhibit strong optical activity attributed to the unique chirality amplification effect [62], rendering them an effective platform for constructing CPL materials with elevated dissymmetry factors [63]. This approach gains significance considering the wide variety of natural and synthetic helical macromolecules available. They reported the successful fabrication of efficient CP-OLEDs using two different chiral helical substituted polyacetylenes, P37 and PSA (Figure 2b), as inducers and an achiral F8BT (Mw = 59 kDa) as the emitter. The resultant CP-OLEDs were fabricated by solution methods, with the active layer prepared from chlorobenzene solutions containing F8BT blended with 25 wt% of R-/S-PSA or P37, followed by annealing at 150 °C for 15 min (Figure 8). The devices benefit from the strong HTP of the chiral helical substituted polyacetylenes and the thermotropic liquid crystal properties of F8BT, achieving a high g_EL_ value of up to 2.0 × 10^−2^ at 545 nm. Furthermore, by employing an inverted device structure ITO/ZnO/F8BT + 25% P37-chiral polymer/TCTA/MoO_3_/Al, the device performance was significantly enhanced, yielding a maximum luminance of 49,340/51,973 cd/m^2^, a high current efficiency of 5.62/5.44 cd/A, and a low turn-on voltage of 3.1/3.1 V, respectively.

### 2.2. PFBT with Chiral Side Chains as Direct CP EL Emitter (Strategy B)

An alternative methodology consists in the direct functionalization of PFBT with chiral side chains, thereby enabling the polymer itself to act as a chiral emitter. By introducing chiral moieties onto the PFBT backbone, researchers have achieved CP emission without the need for external chiral dopants or inductors. Thermal treatment is mandatory and plays a critical role in this approach, as it facilitates the formation of ordered, chiral supramolecular structures that enhance the degree of circular polarization, although it may also adversely affect device performance. This strategy offers improved device stability and reproducibility, making it highly attractive for practical applications.

Abbel et al. [64] introduced chirality into PFBT by incorporating (S)-3,7-dimethyloctyl side chains at the 9-positions of the fluorene monomer. They showed that both the polymer chain length and post-deposition processing are critical factors influencing the structural organization of conjugated polymers in thin films. Notably, the observation of a molecular weight optimum for the chiroptical activity highlights the importance of carefully considering Mw averages and distributions (i.e., polydispersity or Mn/Mw) in the design and optimization of organic polymeric semiconductors [65,66].

**Figure 9:**
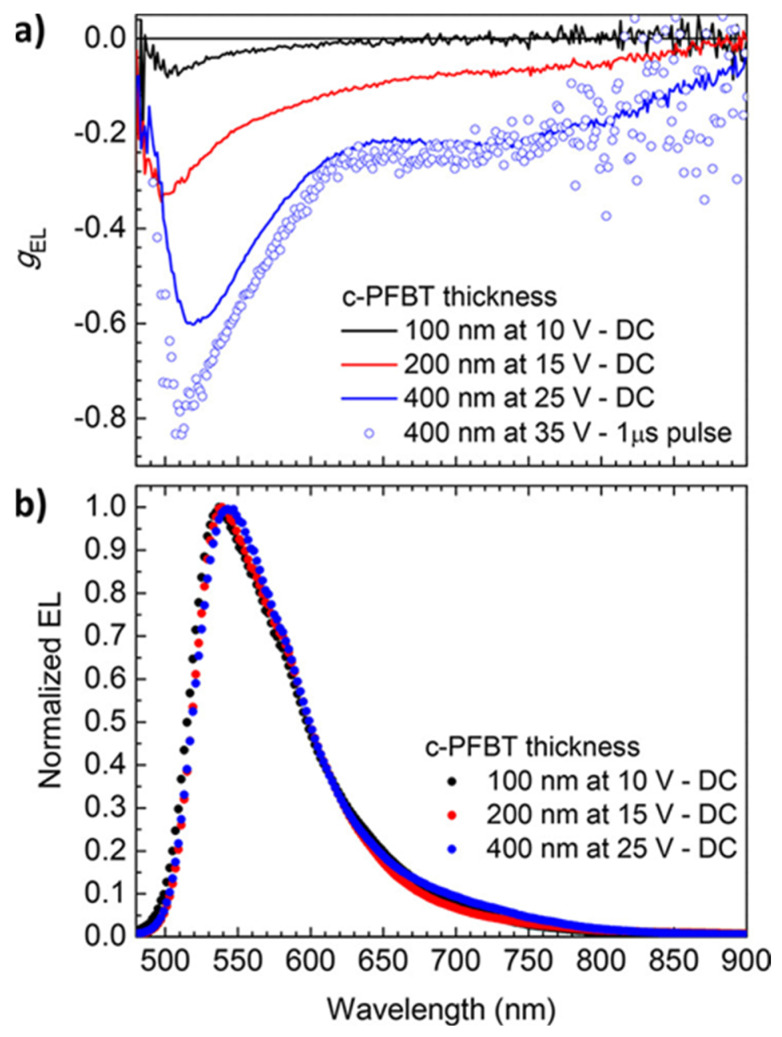
(**a**) g_EL_ of c-PFBT OLEDs of varying active layer thickness. g_EL_ was measured at constant-voltage bias (“DC”, solid lines). g_EL_ measured under 1 μs; 35 V pulsed-voltage excitation (1 kHz repetition rate) on the 400 nm thick OLED is also shown (circles). (**b**) Corresponding total, unpolarized EL spectra under constant-voltage bias. Copyright 2017 American Chemical Society. This publication [67] is licensed under CC-BY.

Di Nuzzo et al. [67] presented a straightforward and effective approach to achieve strong CP EL in single-layer polymer OLEDs by leveraging the self-assembly of an enantiomerically pure chiral PFBT (c-PFBT, Figure 2c) into multidomain cholesteric films, without the need for chiral dopants or alignment layers. The c-PFBT polymer synthesized with specific characteristics (with Mn 15.36 kDa, Mw 31.77 kDa, Mn/Mw = 2.06) features chiral side chains which, upon thermal annealing, organize at the mesoscale into a disordered yet functionally cholesteric structure [64,68]. Efficient CP-OLEDs were obtained only when the c-PFBT emissive layer was thermally annealed at 240 °C prior to the deposition of the top electrode. The pronounced dependence of chiroptical response on thermal history is consistent with the known phase behavior of chirally substituted polyfluorenes: c-PFBT enters the cholesteric liquid–crystalline phase upon heating above 150 °C. Moreover, the magnitude of circular polarization is highly sensitive to film thickness, reflecting the long-range, nonlocal dielectric effects characteristic of cholesteric media on light propagation. By adjusting the thickness of the emitting layer, CP-OLEDs can achieve exceptionally high degrees of CP EL, with g_EL_ ≈ −0.8 under pulsed operation and g_EL_ = −0.6 under constant-voltage bias (Figure 9a), representing some of the highest reported values without utilizing chiral dopants or alignment layers. The study demonstrates that circular polarization primarily arises from nonlocal optical effects occurring after photon generation. As emitted light travels through the cholesteric polymer matrix, circular selective scattering and birefringence convert a portion of the initially LP into CP emission. Experimental analyses, including Mueller matrix ellipsometry and atomic force microscopy imaging, confirm the presence of multidomain cholesteric order with domains a few hundred nanometers in size and strong dielectric anisotropy, which together enable broadband and robust CP EL (Figure 9b). Furthermore, electrical operation parameters, especially the location of the RZ, play a critical role in modulating g_EL_. The relatively large thickness of the cholesteric emitting layer required by this nonlocal approach to strong CP EL, combined with the high annealing temperature (240 °C), presents a significant challenge for achieving high-performance devices: considerable charge injection and light emission in thick structures necessitate high driving voltages (>20 V), consequently restricting device efficiency and stability.

Nonetheless, this work demonstrates that chiral-chain-substituted conjugated polymers can effectively integrate semiconducting behavior with photonic functionality, providing a promising pathway toward efficient, broadband CP-OLEDs. Unfortunately, further development of the chiral PFBT polymer has not been pursued.

A comparative overview of the key chiroptical and electroluminescent figures of merit for CP-OLED systems discussed in the manuscript is reported in Table 1.

## 3. Conclusions and Perspectives

This review establishes F8BT (and c-PFBT) as a central material for CP-OLEDs, owing to the synergistic combination of intrinsically efficient luminescence and their ability to form liquid–crystalline order. These features provide a robust foundation for generating chiral supramolecular structures capable of effectively producing CPL and CP EL. Both approaches examined, chiral induction (in achiral F8BT) via external chiral additives and the integration of chiral side chains (in c-PFBT), demonstrate that the polymer can deliver strong chiroptical responses when its mesoscale organization is appropriately controlled.

Across the reported studies, it becomes evident that the chiroptical properties of F8BT-based systems arise from a subtle interplay between polymer structure and processing conditions. The molecular weight characteristics of the polymer, as well as polydispersity, emerge as decisive parameters governing chain mobility, the formation of a three-dimensional order, and ultimately the magnitude and sign of the circular polarization. Likewise, film-processing variables such as annealing, solvent choice and thickness critically influence supramolecular organization and device behavior.

Notably, helicene-based dopants, such as the aza[6]helicene series blended with F8BT, deliver some of the highest |g_EL_| values in the field, confirming their ability to effectively transfer chirality within the emissive layer. However, when device performance is evaluated through the more integrative Q-factor, a different trend emerges: host–guest strategies such as F8BT:R5011:DBN-ICZ outperform all other materials, despite their more moderate dissymmetry, due to significantly enhanced EQE.

Finally, beyond acting as a chiral emitter, PFBT also proves highly effective as an active host in energy-transfer systems, enabling color tuning and CPL amplification. This versatility underscores its unique position among conjugated polymers and confirms its relevance as a platform for the development of advanced CP-OLED materials and architectures.

Key open challenges concern the deeper understanding and control of the structural parameters that govern chiroptical activity in PFBT-based systems. While the influence of Mw is now recognized, the quantitative relationship between these parameters and supramolecular ordering, helical pitch, and photonic amplification remains largely unresolved. Establishing such correlations will be essential for rationally designing polymers that combine strong CP emission with favorable electronic properties and stable device operation.

A second promising direction involves the refinement of energy-transfer architectures, where PFBT can mediate or amplify CPL through interactions with chiral emitters or chiral host matrices. Further exploration of advanced inducers, such as binaphthalenyl-pyrene derivatives or chiral helical polymers, may enable stronger chirality transfer and higher dissymmetry by introducing rigid, inherently chiral π-systems capable of synergistic interactions with the polymer backbone.

The development of hybrid chiral strategies also represents an exciting frontier. Combining chiral substituted PFBT with external chiral inductors could allow molecular chirality and supramolecular helicity to reinforce one another, offering new routes to tailor birefringence, helical order and recombination zone positioning. Such cooperative approaches may ultimately yield larger g-factors and higher Q-factors at lower driving voltages.

Overall, progress in this field will rely on integrating molecular design, controlled processing and device-level engineering to control the collective phenomena that govern CP EL. Given its unique optical and structural adaptability, PFBT is expected to remain a key material for next-generation CP-OLED technologies across the visible and near-infrared spectrum.

## Figures and Tables

**Table 1:** Summary of EL dissymmetry values and EQEs, where available, and the corresponding Q-factors of PFBT-based CP-OLEDs.

Active Layer	|g_EL_| ^(a)^	EQE (×10^−2^)	Q-Factor (×10^−3^)	Ref.
F8BT:aza[6]H film	0.27	/	/	[37]
F8BT:aza[6]H (110 nm)	0.51	/	/	[49]
F8BT:aza[6]H (160 nm)	1.05	/	/	[49]
F8BT:R-/S-3	~0.01	1.4	~0.014	[58]
F8BT:R/S-6 (5 wt%)	~0.018	0.54	~0.01	[40]
F8BT:R5011:DBN-ICZ	0.16	4.6	7.36	[26]
F8BT:S-P37	0.02	1.2	0.24	[61]
F8BT:S-PSA	0.011	1.51	0.16	[61]
F8BT:R/S5011	1.13	/	/	[29]
F8BT:3TBT-TPA:R5011	0.005	0.28	<0.01	[48]
(S,S)-c-PFBT	0.8	/	/	[67]

^(a)^ best value between the two enantiomers.

## Data Availability

No new data were created or analyzed in this study. Data sharing is not applicable to this article.

## References

[1] Kim Y.-H., Zhai Y., Lu H., Pan X., Xiao C., Gaulding E.A., Harvey S.P., Berry J.J., Vardeny Z.V., Luther J.M. (2021). Chiral-Induced Spin Selectivity Enables a Room-Temperature Spin Light-Emitting Diode. Science.

[2] Farshchi R., Ramsteiner M., Herfort J., Tahraoui A., Grahn H.T. (2011). Optical Communication of Spin Information between Light Emitting Diodes. Appl. Phys. Lett..

[3] Fan H., Li K., Tu T., Zhu X., Zhang L., Liu M. (2022). ATP-Induced Emergent Circularly Polarized Luminescence and Encryption. Angew. Chem. Int. Ed..

[4] Kunnen B., Macdonald C., Doronin A., Jacques S., Eccles M., Meglinski I. (2015). Application of Circularly Polarized Light for Non-Invasive Diagnosis of Cancerous Tissues and Turbid Tissue-like Scattering Media. J. Biophotonics.

[5] Peeters E., Christiaans M.P.T., Janssen R.A.J., Schoo H.F.M., Dekkers H.P.J.M., Meijer E.W. (1997). Circularly Polarized Electroluminescence from a Polymer Light-Emitting Diode. J. Am. Chem. Soc..

[6] Crassous J., Fuchter M.J., Freedman D.E., Kotov N.A., Moon J., Beard M.C., Feldmann S. (2023). Materials for Chiral Light Control. Nat. Rev. Mater..

[7] Geng Y., Trajkovska A., Culligan S.W., Ou J.J., Chen H.M.P., Katsis D., Chen S.H. (2003). Origin of Strong Chiroptical Activities in Films of Nonafluorenes with a Varying Extent of Pendant Chirality. J. Am. Chem. Soc..

[8] Teng J.-M., Zhang D.-W., Wang Y.-F., Chen C.-F. (2022). Chiral Conjugated Thermally Activated Delayed Fluorescent Polymers for Highly Efficient Circularly Polarized Polymer Light-Emitting Diodes. ACS Appl. Mater. Interfaces.

[9] Zinna F., Giovanella U., Bari L.D. (2015). Highly Circularly Polarized Electroluminescence from a Chiral Europium Complex. Adv. Mater..

[10] Fu G., He Y., Li W., Wang B., Lü X., He H., Wong W.-Y. (2019). Efficient Polymer Light-Emitting Diodes (PLEDs) Based on Chiral [Pt(C^N)(N^O)] Complexes with near-Infrared (NIR) Luminescence and Circularly Polarized (CP) Light. J. Mater. Chem. C.

[11] Lu G., Wu Z.-G., Wu R., Cao X., Zhou L., Zheng Y.-X., Yang C. (2021). Semitransparent Circularly Polarized Phosphorescent Organic Light-Emitting Diodes with External Quantum Efficiency over 30% and Dissymmetry Factor Close to 10^−2^. Adv. Funct. Mater..

[12] Brandt J.R., Wang X., Yang Y., Campbell A.J., Fuchter M.J. (2016). Circularly Polarized Phosphorescent Electroluminescence with a High Dissymmetry Factor from PHOLEDs Based on a Platinahelicene. J. Am. Chem. Soc..

[13] Sakai H., Shinto S., Kumar J., Araki Y., Sakanoue T., Takenobu T., Wada T., Kawai T., Hasobe T. (2015). Highly Fluorescent [7]Carbohelicene Fused by Asymmetric 1,2-Dialkyl-Substituted Quinoxaline for Circularly Polarized Luminescence and Electroluminescence. J. Phys. Chem. C.

[14] Song F., Xu Z., Zhang Q., Zhao Z., Zhang H., Zhao W., Qiu Z., Qi C., Zhang H., Sung H.H.Y. (2018). Highly Efficient Circularly Polarized Electroluminescence from Aggregation-Induced Emission Luminogens with Amplified Chirality and Delayed Fluorescence. Adv. Funct. Mater..

[15] Luo X.-F., Han H.-B., Yan Z.-P., Wu Z.-G., Su J., Zou J.-W., Zhu Z.-Q., Zheng Y.-X., Zuo J.-L. (2020). Multicolor Circularly Polarized Photoluminescence and Electroluminescence with 1,2-Diaminecyclohexane Enantiomers. ACS Appl. Mater. Interfaces.

[16] Wan S.-P., Zhao W.-L., Tan K.-K., Lu H.-Y., Li M., Chen C.-F. (2023). Axially Chiral Thermally Activated Delayed Fluorescence Emitters Enabled by Molecular Engineering towards High-Performance Circularly Polarized OLEDs. Chem. Eng. J..

[17] Li M., Chen C.-F. (2022). Advances in Circularly Polarized Electroluminescence Based on Chiral TADF-Active Materials. Org. Chem. Front..

[18] Li M., Chen C.-F. (2026). Highly Efficient Circularly Polarized Electroluminescence Based on a Thermally Activated Delayed Fluorescence Mechanism. Acc. Chem. Res..

[19] Jiang S., Kotov N.A. (2023). Circular Polarized Light Emission in Chiral Inorganic Nanomaterials. Adv. Mater..

[20] Wang Z., Zhao G., Zhang H., Zhou H., Tang Z. (2025). Structural Design and Applications of Chiral Perovskites. Energy Mater. Adv..

[21] Jung E.I., Lee H.J., Kim J., Siddiqui Q.T., Kim M., Lin Z., Park C., Kim D.H. (2024). Recent Progress on Chiral Perovskites as Chiroptical Active Layers for Next-Generation LEDs. Mater. Sci. Eng. R Rep..

[22] Furlan F., Moreno-Naranjo J.M., Gasparini N., Feldmann S., Wade J., Fuchter M.J. (2024). Chiral Materials and Mechanisms for Circularly Polarized Light-Emitting Diodes. Nat. Photonics.

[23] Ji M.-J., Li M., Chen C.-F. (2025). Circularly Polarized Luminescence of Macromolecular Co-Assembly Systems. Chem. Sci..

[24] Zhang D.-W., Li M., Chen C.-F. (2020). Recent Advances in Circularly Polarized Electroluminescence Based on Organic Light-Emitting Diodes. Chem. Soc. Rev..

[25] Liu T., Huang Y. (2025). Circularly Polarized Electroluminescence from Light-Emitting Diodes: Mechanisms, Materials, and Applications. J. Mater. Chem. C.

[26] Guo C.-H., Zhang Y., Zhao W.-L., Tan K.-K., Feng L., Duan L., Chen C.-F., Li M. (2024). Chiral Co-Assembly with Narrowband Multi-Resonance Characteristics for High-Performance Circularly Polarized Organic Light-Emitting Diodes. Adv. Mater..

[27] Arrico L., Di Bari L., Zinna F. (2021). Quantifying the Overall Efficiency of Circularly Polarized Emitters. Chem.—Eur. J..

[28] Xu Q., Fu J., Tang M., Yao H., Lin J. (2025). Circularly Polarized Luminescence in Chiral Materials: Navigating Trade-Offs between Luminescence Dissymmetry Factor and Photoluminescence Quantum Yield. Adv. Opt. Mater..

[29] Lee D.-M., Song J.-W., Lee Y.-J., Yu C.-J., Kim J.-H. (2017). Control of Circularly Polarized Electroluminescence in Induced Twist Structure of Conjugate Polymer. Adv. Mater..

[30] Kulkarni C., Van Son M.H.C., Di Nuzzo D., Meskers S.C.J., Palmans A.R.A., Meijer E.W. (2019). Molecular Design Principles for Achieving Strong Chiroptical Properties of Fluorene Copolymers in Thin Films. Chem. Mater..

[31] Vohra V., Mróz W., Inaba S., Porzio W., Giovanella U., Galeotti F. (2017). Low-Cost and Green Fabrication of Polymer Electronic Devices by Push-Coating of the Polymer Active Layers. ACS Appl. Mater. Interfaces.

[32] De Brito E.B., Santos D.C., De Paula T.P., De Morais A., De Freitas J.N., Valaski R., Marques M.D.F.V., Cocca L.H.Z., Pelosi A.G., De Boni L. (2024). Synthesis and Characterization of Novel Fluorene–Based Green Copolymers and Their Potential Application in Organic Light-Emitting Diodes. J. Mater. Res. Technol..

[33] Squeo B.M., Mróz W., Giovanella U., Pasini M. (2020). Anionic Low Band Gap-Conjugated Polyelectrolytes as Hole-Transporting Layer in Optoelectronics Devices. Chem. Proc..

[34] Zhang Y., Yu S., Han B., Zhou Y., Zhang X., Gao X., Tang Z. (2022). Circularly Polarized Luminescence in Chiral Materials. Matter.

[35] Zhong H., Gao X., Zhao B., Deng J. (2024). “Matching Rule” for Generation, Modulation and Amplification of Circularly Polarized Luminescence. Acc. Chem. Res..

[36] Han J., Guo S., Lu H., Liu S., Zhao Q., Huang W. (2018). Recent Progress on Circularly Polarized Luminescent Materials for Organic Optoelectronic Devices. Adv. Opt. Mater..

[37] Yang Y., da Costa R.C., Smilgies D.-M., Campbell A.J., Fuchter M.J. (2013). Induction of Circularly Polarized Electroluminescence from an Achiral Light-Emitting Polymer via a Chiral Small-Molecule Dopant. Adv. Mater..

[38] Dhbaibi K., Abella L., Meunier-Della-Gatta S., Roisnel T., Vanthuyne N., Jamoussi B., Pieters G., Racine B., Quesnel E., Autschbach J. (2021). Achieving High Circularly Polarized Luminescence with Push–Pull Helicenic Systems: From Rationalized Design to Top-Emission CP-OLED Applications. Chem. Sci..

[39] Luo X.-F., He J., Wang Y., Dai H., Wu Z.-G. (2022). Research Advances in Helicene Structure-Based Chiral Luminescent Materials and Their Circularly Polarized Electroluminescence. Chin. J. Struct. Chem..

[40] Zhang X., Xu Z., Zhang Y., Quan Y., Cheng Y. (2021). Controllable Circularly Polarized Electroluminescence Performance Improved by the Dihedral Angle of Chiral-Bridged Binaphthyl-Type Dopant Inducers. ACS Appl. Mater. Interfaces.

[41] Sugiura K. (2020). [2.2]Paracyclophane-Based Chiral Platforms for Circularly Polarized Luminescence Fluorophores and Their Chiroptical Properties: Past and Future. Front. Chem..

[42] Sánchez-Carnerero E.M., Agarrabeitia A.R., Moreno F., Maroto B.L., Muller G., Ortiz M.J., de la Moya S. (2015). Circularly Polarized Luminescence from Simple Organic Molecules. Chem.—Eur. J..

[43] Mori T. (2026). Small Molecule Helical Emitters. Chem. Soc. Rev..

[44] Sang Y., Han J., Zhao T., Duan P., Liu M. (2020). Circularly Polarized Luminescence in Nanoassemblies: Generation, Amplification, and Application. Adv. Mater..

[45] Wade J., Hilfiker J.N., Brandt J.R., Liirò-Peluso L., Wan L., Shi X., Salerno F., Ryan S.T.J., Schöche S., Arteaga O. (2020). Natural Optical Activity as the Origin of the Large Chiroptical Properties in π-Conjugated Polymer Thin Films. Nat. Commun..

[46] Donley C.L., Zaumseil J., Andreasen J.W., Nielsen M.M., Sirringhaus H., Friend R.H., Kim J.-S. (2005). Effects of Packing Structure on the Optoelectronic and Charge Transport Properties in Poly(9,9-di-*n*-Octylfluorene-*alt*-Benzothiadiazole). J. Am. Chem. Soc..

[47] Gust D., Scholz M., Schumacher V., Mulatier J.-C., Pitrat D., Guy L., Oum K., Lenzer T. (2024). Annealing Temperature-Dependent Induced Supramolecular Chiroptical Response of Copolymer Thin Films Studied by Pump-Modulated Transient Circular Dichroism Spectroscopy. Sci. Rep..

[48] Squeo B.M., Arrigoni A., Zinna F., Di Bari L., Botta C., Pasini M., Giovanella U. (2025). Near-Infrared Electroluminescent Conjugated Copolymer: Triphenyalmine-Functionalized Benzothiadiazole-Thiophene System for Circularly Polarized OLEDs. Macromol. Rapid Commun..

[49] Wan L., Wade J., Salerno F., Arteaga O., Laidlaw B., Wang X., Penfold T., Fuchter M.J., Campbell A.J. (2019). Inverting the Handedness of Circularly Polarized Luminescence from Light-Emitting Polymers Using Film Thickness. ACS Nano.

[50] Stará I.G., Starý I. (2022). Synthesis of Helicenes by [2 + 2 + 2] Cycloisomerization of Alkynes and Related Systems. Helicenes.

[51] Rodriguez R., Del Rio N., Crassous J. (2022). Organometallic and Coordination Chemistry of Helicenes. Helicenes.

[52] Ryan S.T.J., Fuchter M.J. (2022). Helicenes for Optoelectronic Applications and Devices. Helicenes.

[53] Shen Y., Chen C.-F. (2012). Helicenes: Synthesis and Applications. Chem. Rev..

[54] Hassey R., Swain E.J., Hammer N.I., Venkataraman D., Barnes M.D. (2006). Probing the Chiroptical Response of a Single Molecule. Science.

[55] Gust D., Morgenroth M., Scholz M., Schumacher V., Mulatier J.-C., Pitrat D., Guy L., Oum K., Lenzer T. (2025). Unexpected Sign Inversion of the Circular Dichroism and Circularly Polarized Luminescence Response of Chiral Copolymer Thin Films by Tuning the Thickness and Annealing Conditions. ChemPhotoChem.

[56] Giovanella U., Pasini M., Botta C., Bergamini G., Silvi S. (2016). Organic Light-Emitting Diodes (OLEDs): Working Principles and Device Technology. Applied Photochemistry: When Light Meets Molecules.

[57] Yan H., Wade J., Wan L., Kwon S., Fuchter M.J., Campbell A.J., Kim J.-S. (2022). Enhancing Hole Carrier Injection via Low Electrochemical Doping on Circularly Polarized Polymer Light-Emitting Diodes. J. Mater. Chem. C.

[58] Zhang X., Xu Z., Zhang Y., Quan Y., Cheng Y. (2020). High Brightness Circularly Polarized Electroluminescence from Conjugated Polymer F8BT Induced by Chiral Binaphthyl-Pyrene. J. Mater. Chem. C.

[59] Wan L., Wade J., Shi X., Xu S., Fuchter M.J., Campbell A.J. (2020). Highly Efficient Inverted Circularly Polarized Organic Light-Emitting Diodes. ACS Appl. Mater. Interfaces.

[60] Furlan F., Šámal M., Rybáček J., Taddeucci A., Di Girolamo M., Nodari D., Siligardi G., Wade J., Yan B., Stará I.G. (2025). Electrical Control of Photon Spin Angular Momentum in Organic Electroluminescent Materials. Nat. Photonics.

[61] Wang M., Yang K., Wang X., Tan Z., Pan K., Deng J., Zhao B. (2024). Chiral Helical Polymer-Induced Efficient Circularly Polarized Organic Light-Emitting Diodes. Adv. Opt. Mater..

[62] Wu Z.-Q., Song X., Li Y.-X., Zhou L., Zhu Y.-Y., Chen Z., Liu N. (2023). Achiral Organoiodine-Functionalized Helical Polyisocyanides for Multiple Asymmetric Dearomative Oxidations. Nat. Commun..

[63] Li S.-Y., Xu L., Gao R.-T., Chen Z., Liu N., Wu Z.-Q. (2023). Advances in Circularly Polarized Luminescence Materials Based on Helical Polymers. J. Mater. Chem. C.

[64] Abbel R., Schenning A.P.H.J., Meijer E.W. (2008). Molecular Weight Optimum in the Mesoscopic Order of Chiral Fluorene (Co)Polymer Films. Macromolecules.

[65] Kulkarni C., Meskers S.C.J., Palmans A.R.A., Meijer E.W. (2018). Amplifying Chiroptical Properties of Conjugated Polymer Thin-Film Using an Achiral Additive. Macromolecules.

[66] Kulkarni C., Di Nuzzo D., Meijer E.W., Meskers S.C.J. (2017). Pitch and Handedness of the Cholesteric Order in Films of a Chiral Alternating Fluorene Copolymer. J. Phys. Chem. B.

[67] Di Nuzzo D., Kulkarni C., Zhao B., Smolinsky E., Tassinari F., Meskers S.C.J., Naaman R., Meijer E.W., Friend R.H. (2017). High Circular Polarization of Electroluminescence Achieved via Self-Assembly of a Light-Emitting Chiral Conjugated Polymer into Multidomain Cholesteric Films. ACS Nano.

[68] Lakhwani G., Meskers S.C.J. (2012). Insights from Chiral Polyfluorene on the Unification of Molecular Exciton and Cholesteric Liquid Crystal Theories for Chiroptical Phenomena. J. Phys. Chem. A.

